# Gap junctions: historical discoveries and new findings in the *Caenorhabditis* *elegans* nervous system

**DOI:** 10.1242/bio.053983

**Published:** 2020-09-03

**Authors:** Eugene Jennifer Jin, Seungmee Park, Xiaohui Lyu, Yishi Jin

**Affiliations:** Neurobiology Section, Division of Biological Sciences, University of California, San Diego, La Jolla, CA 92093, USA

**Keywords:** Innexins, Neuronal development, Circuit wiring and rewiring, Locomotion, Chemosensory response, Noxious response, Neural circuit, Stress condition, Dauer

## Abstract

Gap junctions are evolutionarily conserved structures at close membrane contacts between two cells. In the nervous system, they mediate rapid, often bi-directional, transmission of signals through channels called innexins in invertebrates and connexins in vertebrates. Connectomic studies from *Caenorhabditis elegans* have uncovered a vast number of gap junctions present in the nervous system and non-neuronal tissues. The genome also has 25 innexin genes that are expressed in spatial and temporal dynamic pattern. Recent findings have begun to reveal novel roles of innexins in the regulation of multiple processes during formation and function of neural circuits both in normal conditions and under stress. Here, we highlight the diverse roles of gap junctions and innexins in the *C. elegans* nervous system. These findings contribute to fundamental understanding of gap junctions in all animals.

## Abstract (precis)

**Summary:** Studies from *C. elegans* have illustrated many roles of gap junctions in neural connectivity and circuit regulation, during normal conditions and under stress.

## Introduction

In all animals, neurons transmit signals through two main types of synaptic connections: chemical synapses and electrical synapses, the latter also known as gap junctions. Chemical synapses are morphologically and functionally asymmetric junctions formed between a neuron and its targets (neuron or muscle). The presynaptic terminal is characterized by an aggregation of neurotransmitter-filled synaptic vesicles around the electron-dense membranes, whereas the postsynaptic side contains receptors for neurotransmitters and signal transduction molecules. Pre- and postsynaptic membranes are separated by a 20–30 nm wide extracellular space, known as the synaptic cleft, which is generally wider than non-synaptic space between two adjacent neurons and consists of specialized extracellular matrix and signaling molecules to facilitate milli-second transmission of neural information (Ahmari and Smith, 2002; Goyal and Chaudhury, 2013; Südhof, 2018). In contrast, electrical synapses or gap junctions are morphologically symmetrical, defined by two electron-dense membranes that can be as narrow as 2–3 nm apart (Goodenough and Paul, 2009; Miller and Pereda, 2017). In a gap junction, two cells are connected through channels that allow free exchange of small molecules such as ions and second messenger molecules below ∼1 kDa (Goodenough, 1996). Both chemical and electrical synaptic transmission are required for normal brain development and function. Chemical synaptic transmission is well studied in neuronal activity and nervous system function. While gap junctions are generally thought to transmit signals symmetrically between partner cells and regulate the neuronal circuit in coordination with chemical synapses, it is also becoming evident that gap junction proteins have diverse roles in neuronal development and function, and tissue communication under stress conditions, as both channels and hemichannels (Evans, 2015; Hasegawa and Turnbull, 2014; Güiza et al., 2018; Miller and Pereda, 2017; Oshima, 2019; Altun et al., 2009).

**Fig. 1.**
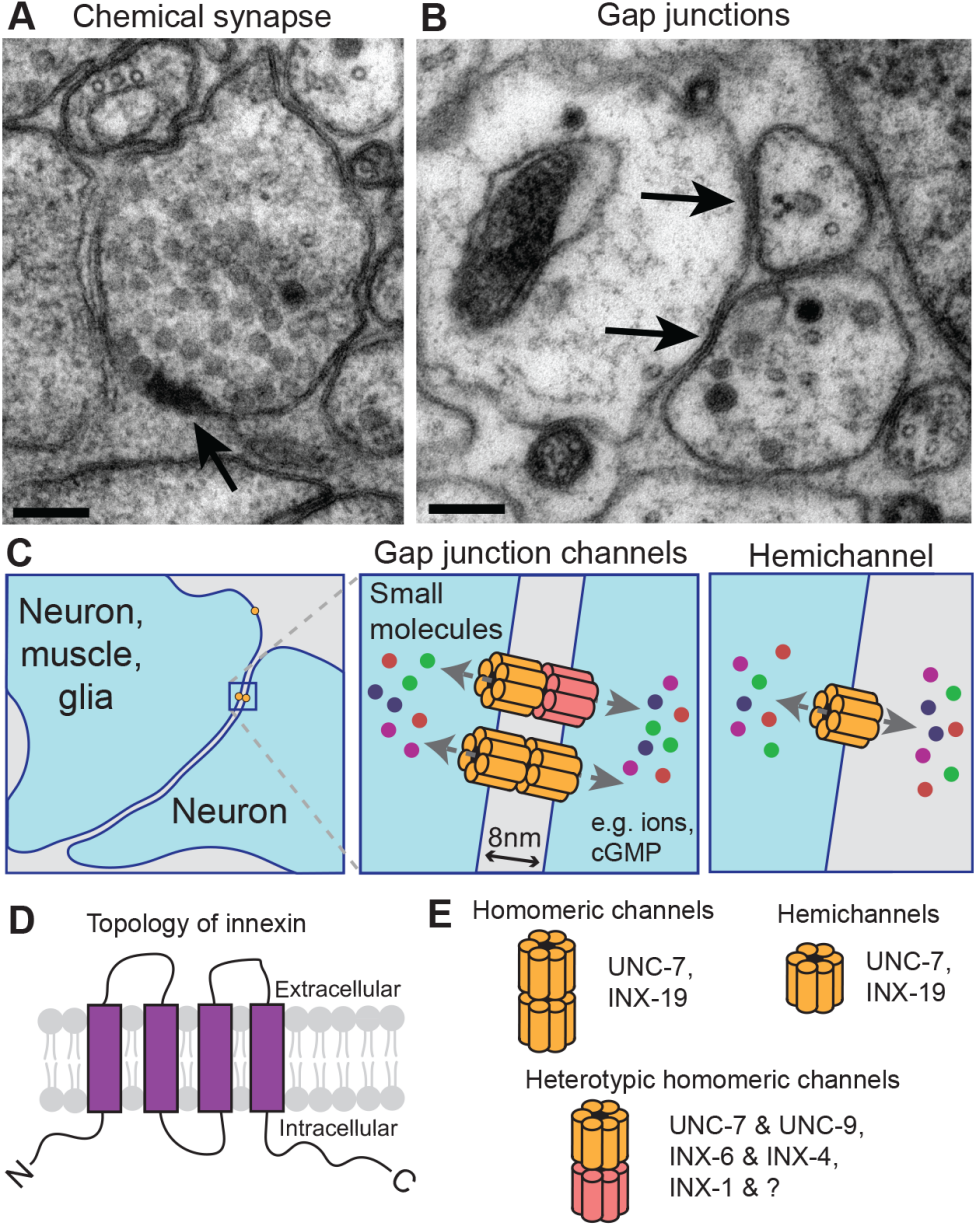
**Gap junction and gap** **junction channels in the *C. elegans* nervous system.** (A,B) Electron micrograph (EM) images of chemical and electrical synapses, or gap junctions, in the *C. elegans* ventral nerve cord. Arrows indicate chemical synapse in (A) and gap junctions in (B). Scale bars in (A) and (B): 100 nm. (C) Illustration of gap junction channels and hemichannels in the *C. elegans* nervous system. Neurons form gap junctions with other neurons, muscles and glia. Gap junction channels are composed of two hemichannels, and these allow direct exchange of small molecules such as ions and cGMP (colorful small circles) between two cells (light blue). A gap of about 8 nm separating two membranes was reported in EM reconstruction (White et al., 1986). Hemichannels allow ion transfer between neuron (blue) and extracellular space (grey). Each hemichannel is composed of six or eight Innexins. (D) Gap junction proteins, Innexins, contain four transmembrane domains and intracellular N- and C-termini. Although sequence similarity between innexins and vertebrate gap junction proteins, connexins, is low, the topology of innexins and connexins are conserved. (E) Gap junction channel types in *C. elegans* nervous system. Homomeric channels are composed of two hemichannels of the same innexin protein. Heterotypic homomeric channels are composed of two different hemichannels. Each hemichannel is illustrated as composed of six subunits, but some may exist in eight subunits (e.g. INX-6).

The nematode *Caenorhabditis elegans* is a small (1 mm long as adult), optically transparent and genetically tractable model organism with a generation time of 3–4 days. Pioneering work completed nearly 40 years ago led to the complete reconstruction of nervous system connectivity at the ultrastructural level (White et al., 1986). The chemical and electrical synaptic connectivity are well characterized at both ultrastructural and cellular resolutions (Fig. 1A,B) (White et al., 1976, 1986; Jarrell et al., 2012). In adult hermaphrodites, the nervous system consists of 302 neurons, with connections to other cell types such as muscles, glial cells and epidermal cells. With the recent addition of sex-specific and developmental connectomic data, the nervous system is estimated to have 8000 chemical synapses and close to 2000 gap junctions (Witvliet et al., 2020; Cook et al., 2019). While chemical synapses typically form between neurons or between neurons and muscles, gap junctions are present between neurons as well as non-neuronal cells such as muscles and glia (Fig. 1C) (White et al., 1986, 1976; Hall, 2019). Together with powerful molecular genetic tools, the advantages of *C. elegans* as a model system allow characterization of gap junctions and gap junction proteins at genetic, molecular, cellular, ultrastructural and behavioral levels (Hall, 2019). Recent functional analyses of *C. elegans* gap junctions in the nervous system demonstrate their functional equivalence to mammalian gap junctions, highlighting that studies in *C. elegans* contribute to our fundamental understanding of how gap junctions regulate brain development and function. Here, we provide an overview of the historical studies of gap junction genes in *C. elegans*, then describe key recent findings on the various roles of gap junctions in neuronal development, circuit regulation and under stress conditions.

## Discovery of innexin genes

The first gap junction gene characterized in *C. elegans* was *unc-7*, identified in the very first genetic screen conducted by Sydney Brenner, (Brenner, 1974). Wild type *C. elegans* exhibit smooth sinusoidal locomotion, and mutants with abnormal movement were named as uncoordinated (*unc*). *unc* mutants were later grouped into phenotypic subclasses (Hodgkin, 1983). *unc-7* belongs to the ‘forward uncoordinated’ class, nicknamed ‘kinkers’ (Yeh et al., 2009). Early on, it was noted that the severe locomotion defects of *unc-7* mutants were likely associated with abnormal gap junctions in the motor circuit (Starich et al., 1993). In wild type animals, cholinergic B-type motor neurons (DB and VB) receive input from premotor interneuron AVB, not AVA, but in *unc-7* mutants B-type neurons appear to form gap junctions with AVA (J. G. White, personal communication documented in Starich et al., 1993). Starich et al. (1993) cloned *unc-7* and reported that the predicted protein has four transmembrane domains (Fig. 1D). Later studies showed UNC-7 is related to *Drosophila* gap junction proteins Passover, Ogre and Shaking-B (Barnes and Hekimi, 1997).

Two more gap junction genes, *unc-9* and *eat-5*, were cloned based on amino acid sequence similarity to UNC-7 and the *Drosophila* gap junction proteins. *unc-9* mutants were also first identified by Brenner, and displayed the same locomotion and egg-laying defects as *unc-7* mutants, which led to the proposal that these two genes probably function together in establishing electrical connectivity in the nervous system (Barnes and Hekimi, 1997). *eat-5* mutants were identified based on pharynx pumping defects due to lack of electrical coupling between the pharyngeal muscles based on dye-coupling assay (Avery, 1993; Starich et al., 1996). The pharyngeal pumping defects in *eat-5* mutants can be suppressed by increasing the normally functionally silenced chemical synaptic transmission from motor neuron M4 to terminal bulb pharyngeal muscle in *slo-1* (BK calcium-activated potassium channel) or *unc-2/unc-36* (voltage gated calcium channel) mutants (Steciuk et al., 2014; Chiang et al., 2006). These observations highlight the importance of understanding both chemical as well as electrical connectivity in the nervous system.

In the late 1990s, the completion of *C. elegans* genome sequences, along with PCR screening based on nucleotide sequence homology, led to the identification of 25 *C. elegans* genes in the OPUS family (Starich et al., 1996; C. elegans Sequencing Consortium, 1998). Immunocytochemical staining using UNC-7 and EAT-5 antibodies confirmed the proteins localized to the plasma membrane (Starich et al., 2001). Thus, these and other invertebrate gap junction genes were named innexins (invertebrate connexins) or *inx* (Phelan et al., 1998). By amino acid sequence innexins show no scorable similarities to connexins, the vertebrate gap junction proteins (Phelan et al., 1998; Skerrett and Williams, 2017). However, the presence of four transmembrane proteins with predicted membrane topology as well as the formation of both gap junction channels and hemichannels by innexins make innexins and connexions into functionally equivalent molecules (Fig. 1D,E). As discussed later, this idea has received support from emerging experimental evidence.

## Expression of innexins is broad and dynamic with cell-type specificity

The expression patterns for all *C. elegans* innexins were determined using a series of approaches. Transcriptional reporters of innexins provided early insights into cell type specificity (Altun et al., 2009; Starich et al., 2001). Recently, fosmid-based gene-tagging has enabled the identification of all innexin-expressing neurons (Bhattacharya et al., 2019). While none of the innexins is pan-neuronally expressed, many innexins (*inx-1*, *inx-2*, *che-7*, *inx-7*, *inx-10*, *inx-14*, *inx-18*, *inx-19*, *unc-7* and *unc-9*) are broadly expressed in the nervous system. A given neuron expresses at least one innexin, with most neurons generally expressing seven to nine innexins. Other *inx* genes show restricted expression, for example, *inx-6* and *eat-5* show exclusive expression in only one neuron type. About ten innexins (*inx-3*, *inx-8*, *inx-9*, *inx-12*, *inx-15*, *inx-16*, *inx-17*, *inx-20*, *inx-21* and *inx-22*) are non-neuronally expressed. Regulation at the levels of transcription or mRNA splicing further contributes to *inx* gene expression complexity. For example, two different isoforms of innexins can have similar (*inx-1a* and *inx-1b*) or different (*inx-18a* and *inx-18b*) expression patterns, and the *inx-10a* isoform is only expressed in I6 neuron. The expression profiling data provide a foundation for investigating the roles of innexins in the nervous system. For example, *eat-5* and *inx-3* have partially overlapping expression in the pharynx, but likely function independently, since when expressed separately in the two oocytes they do not form intercellular channels between paired *Xenopus* oocytes (Landesman et al., 1999; Starich et al., 1996). Using the same assay, INX-3 but not EAT-5 was found to form homomeric channels when expressed in both oocytes. *inx-6* also shares similar pharyngeal expression as *eat-5*, and *inx-6* mutant phenotypes are partially rescued by *eat-5* expression with *inx-6* promoter (Li et al., 2003).

## Innexins have many roles in the nervous system

Functional studies of *C. elegans* innexins have been driven by a combination of phenotype-based forward genetic screens and systematic characterization of genetic knockout mutations. These studies have shown complex functional redundancy and independence. Compared to non-neuronal systems such as the pharynx, body wall muscle coupling and germ line development in *C. elegans*, the roles of innexins in the nervous system are less well understood (Hall, 2017; Simonsen et al., 2014). Below, we highlight several examples of functional studies of innexins in the *C. elegans* nervous system.

### Olfactory neuron fate specification

AWC olfactory neurons are a bilaterally symmetric pair of morphologically identical cells in the anterior of the head. Yet, they are molecularly and functionally asymmetric, distinguished by their expression of the G-protein coupled receptor (GPCR) STR-2 as AWC^on^ or AWC^off^ (Wes and Bargmann, 2001; Bauer Huang et al., 2007). The specification of AWC^on^ vs AWC^off^ occurs in late embryogenesis in a stochastic manner such that 50% of a population expresses STR-2, a GPCR, in the left-side neuron while the other half expresses STR-2 in the right-side neuron (Troemel et al., 1999). The differential expression of STR-2 results in functional asymmetry between the two AWC neurons.

**Fig. 2.**
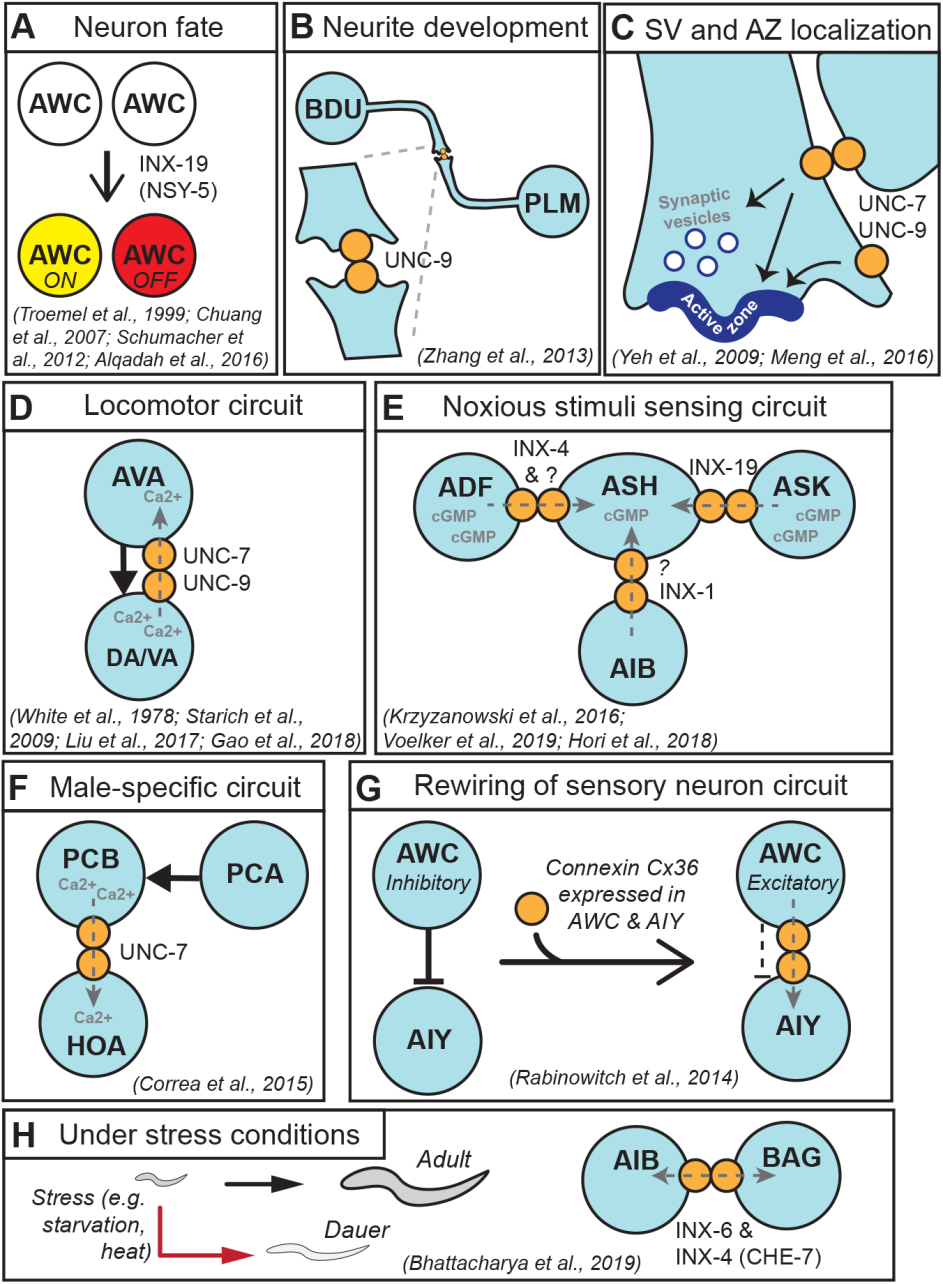
**Gap junctions and innexins have diverse roles in the *C. elegans* nervous system.** (A) *inx-19* (*nsy-5*) is required for AWC neurons’ cell fate differentiation for left–right asymmetry. (B) UNC-9 localizes to the contact site between BDU and PLM neurites. (C) UNC-7 and UNC-9 form gap junctions between RME neuron and GLR and are required for SV and active zone protein localization. UNC-7 hemichannels on D-type motor neurons are required for active zone protein localization. (D) In the locomotor circuit, AVA interneurons and A-type motor neurons (DA/VA) form both chemical synapses and gap junctions. Chemical input (black solid arrow) is from AVA to DA/VA. Gap junctions are formed by UNC-7 on AVA and UNC-9 on DA/VA, and allow antidromic rectifying current (grey dashed arrow). (E) ASH neurons sense noxious stimuli and are connected to ADF, ASK and AIB neurons via gap junctions composed of INX-4, INX-19 and INX-1, respectively. cGMP transfers through the gap junctions into ASH neurons, dampens calcium levels in ASH neurons and leads to reduced quinine sensitivity. (F) In the male-specific circuit, PCA chemically synapses onto PCB. PCB and HOA form UNC-7-dependent gap junctions that allow calcium transfer from PCB to HOA. (G) AWC forms inhibitory chemical synapse with AIY. Ectopic expression of vertebrate gap junction gene Cx36 in AWC and AIY results in gap junction formation and excitatory synaptic connectivity between AWC and AIY. (H) Under stressful environmental condition such as starvation, heat and high population density, young larvae undergo stress resistant dauer arrest. Dauer nervous system expresses dauer-specific and non-dauer-specific gap junction proteins. AIB neurons express INX-6 only in dauer, to form gap junction with BAG neuron that expresses INX-4 (CHE-7) in both dauer and non-dauer.

Genes required for asymmetry of AWCs were identified in a genetic screen for ‘*nsy (neuronal symmetry)*’ mutants showing loss of asymmetric STR-2 expression in AWC. For example, loss of function in *nsy-5* causes both AWC neurons to differentiate into the AWC^off^ subtype (Chuang et al., 2007). *nsy-5* encodes INX-19, expressed in both AWC neurons and at least 17 other pairs of neighboring neurons. Ultrastructural analysis reveals that AWCs and some adjacent neurons form an extensive network of gap junctions during embryogenesis when expression of NSY-5/INX-19 peaks. The extensive network of neurons involving NSY-5/INX-19 gap junctions has been shown to be critical for intercellular calcium signaling in establishing the asymmetry of AWCs (Schumacher et al., 2012) (Fig. 2A). The symmetric AWC^off^ phenotype in *nsy-5/inx-19* mutants can be completely suppressed by a gain-of-function mutation of the SLO-1 voltage and calcium-activated large conductance BK potassium channel, suggesting that *slo-1* acts downstream of *nsy-5* (Alqadah et al., 2016). *nsy-5/inx-19* is important for asymmetric expression of *slo-1* in AWC neurons. Interestingly, NSY-5 /INX-19 can also function as a hemichannel as well as gap junction (Chuang et al., 2007). It is not yet known how and which function of NSY-5/INX-19 is critical for the asymmetric expression of the downstream SLO-1 for proper differentiation of AWCs into the two functionally and molecularly distinct subtypes.

### Mechanosensory neurite development

Mechanosensory neuron PLM and interneuron BDU are physically connected to each other, and form innexin UNC-9 clusters at their interface only upon contact (Zhang et al., 2013) (Fig. 2B). To understand how the PLM-BDU physical connection was regulated, Zhang et al. investigated multiple signaling molecules, and found select components of the Wnt pathway to be critical for the contact. Moreover, they performed a forward genetic screen for mutants exhibiting PLM-BDU contact defects, and found *aha-1*, the sole *C. elegans* ortholog of ARNT (AHR nuclear translocator, containing a basic helix-loop-helix domain), to be required in both PLM and BDU for the proper contact between them. Expression of *cam-1*, which encodes a Wnt receptor necessary for the PLM-BDU contact, was significantly reduced in *aha-1* mutants. It remains to be determined whether UNC-9 localization at PLM-BDU contact site also requires Wnt signaling and AHA-1. This study demonstrates that tight regulation of gap junction formation between neurons is important for proper neurite formation.

### Regulation of chemical synapses

Electrical synapses are often present near chemical synapses, raising the possibility that their localizations may be co-regulated (Jabeen and Thirumalai, 2018). A defining feature of chemical synapses is the accumulation of synaptic vesicles (SV) at the neurotransmitter release site, the ‘active zone’. The Liprin-α protein SYD-2 is a central organizer for active zone formation (Zhen and Jin, 1999). In a forward genetic screen using GFP-tagged SYD-2 as reporter, innexin UNC-7 was identified to be important for active zone protein localization in GABAergic D-type motor neurons (Yeh et al., 2009). The role of UNC-7 in regulation of active zone morphology was not restricted to GABAergic motor neurons. Ultrastructural analysis revealed that active zone morphology was abnormal in both GABAergic and cholinergic motor neurons of *unc-7* mutants. Despite its impact on the active zone, UNC-7 was not critical for SV distribution. The closely related innexin UNC-9 was also shown to be critical for active zone protein localization. UNC-7 and UNC-9 colocalized at perisynaptic regions adjacent to active zone protein or SV proteins. However, gap junctions are not present near active zones in motor neurons at the ultrastructural level (White et al., 1986). The authors suggest that the cell-autonomous role of UNC-7 in active zone formation is likely independent of its gap junction channel function.

In another forward genetic screen for genes regulating synapse position of GABAergic RME neurons, a gap junctional role of UNC-7 was found to be critical for both SV and active zone localization (Meng et al., 2016) (Fig. 2C). RME neurons form gap junctions with neighboring glial cells, GLR (White et al., 1986). Meng et al. identified *unc-1*, a homolog of Stomatin (Rajaram et al., 1998) that is a regulator of gap junction function (Chen et al., 2007), to be critical for proper SV localization. *unc-1* function is required in both RME neurons and GLR cells for SV localization. Both *unc-7* and *unc-9* mutants exhibited altered SV localization in RME neurons. The chemical synaptic phenotype of *unc-7* mutants was fully rescued only when UNC-7 was expressed in both RME neurons and GLR cells. Moreover, expression of a gap-junction defective mutant form of UNC-7 failed to rescue the phenotype. These findings demonstrate that the junctional role of UNC-7 is crucial to SV localization in RME neurons (Fig. 2C). Similarly in the vertebrate nervous system, gap junctional communication regulates chemical synapse formation and connectivity (Peinado et al., 1993), demonstrating functional equivalence of innexins to connexins.

## Important roles of innexins in many neural circuits

An outstanding question in understanding innexins and gap junctions is ‘what molecules are transported through gap junctions?’ Here, we describe recent discoveries on the identification of the small molecules that pass through innexin channels in the *C. elegans* nervous system, and how these regulate various neural circuits. These examples provide evidence that innexins have functionally equivalent gap junctional roles to connexins in regulating neuronal activity.

### Antidromic-rectifying current in locomotor circuit

In further classification of Brenner's uncoordinated mutants, Hodgkin was the first to group *unc-7* and *unc-9* together under the descriptive category of ‘no mating and forward uncoordinated’ (Hodgkin, 1983). *unc-7* and *unc-9* were also identified as suppressors of *unc-79* and *unc-80* mutants’ increased sensitivity to volatile anesthetics (Sedensky and Meneely, 1987; Morgan et al., 1990, 1991). It was later found that UNC-7 and UNC-9 indeed function together by forming homomeric and heterotypic gap junctions in the motor circuit (Starich et al., 2009; Yeh et al., 2009).

In the locomotor circuit, the A-type (DA and VA) and B-type (DB and VB) motor neurons are connected to interneurons AVA and AVB, respectively, via *unc-7* and *unc-9* dependent gap junctions (Starich et al., 2009; Liu et al., 2017). Intracellular recording of VA5 neuron revealed that bursts in post synaptic current (PSC) in VA5 require *unc-7* and *unc-9* in AVA and A-motor neurons, respectively (Liu et al., 2017). In wild type animals, PSC bursts in VA5 coincided with calcium transients and PSC bursts in adjacent body wall muscle cells. Injecting current into VA5 resulted in corresponding current changes in AVA, but not vice versa, and this electrical coupling required *unc-7* and *unc-9*. Spontaneous PSCs that resulted from chemical synaptic input from AVA remained unaltered in *unc-7* and *unc-9* mutant backgrounds. The authors proposed that the gap junctions provide an antidromic rectifying current (from VA to AVA, opposite to the chemical synaptic transmission from AVA to VA) and amplify the spontaneous activity in VA into PSC bursts (Fig. 2D). At rest, however, loss of *unc-7* caused increased calcium oscillation in DA9 neurons compared to wild type or chemical synaptic transmission mutant *unc-13* animals (Gao et al., 2018). These results suggest that the neuromodulatory effects of gap junctions depend on the activity state of neurons.

### Second messenger cGMP in noxious stimuli sensing circuit

ASH sensory neurons are the primary nociceptors responsible for quinine avoidance response. ASH is electrically connected to two other sensory neurons AFD, ADF, and interneuron AIA neurons via INX-4 (aka CHE-7) (Krzyzanowski et al., 2016), and also to ASK sensory neurons via homomeric INX-19 (aka NSY-5) gap junctions (Voelker et al., 2019) (Fig. 2E). cGMP traffics through these gap junctions and modulate quinine sensitivity. Optogenetic stimulation of cGMP production using blue light-inducible guanylyl cyclase (Ryu et al., 2010) in ADF neurons resulted in reduced quinine sensitivity, but not in *inx-4* mutant, suggesting that cGMP transfer from ADF to ASH neurons dampens quinine sensitivity (Krzyzanowski et al., 2016). When endogenous cGMP level was measured using a fluorescent reporter FlincG3 (Woldemariam et al., 2019; Bhargava et al., 2013), loss of *inx-19* resulted in increased cGMP levels in ASK neurons and reduced cGMP levels in ASH neurons (Voelker et al., 2019). However, calcium level was increased in ASH neurons in *inx-19* mutant, suggesting that increased cGMP flow from ASK to ASH neurons dampens calcium levels in the ASH neurons, resulting in reduced quinine sensitivity (Voelker et al., 2019).

*C. elegans* show three types of nociceptive avoidance behaviors: long turn, short turn and omega turn. Weak sorbitol stimuli result in higher probability of short or long reversal behaviors, while strong sorbitol stimuli often result in omega turn avoidance behavior (Hori et al., 2018). In a screen for regulators of these distinct avoidance behaviors, Hori et al. identified the transcription factor *lin-32* to be required for omega-turn behavior in response to high optogenetic stimulation of ASH neurons or noxious stimuli (Hori et al., 2018). *lin-32* is required for AIB interneuron differentiation including INX-1 expression. *inx-1* mutants display reduced probability of omega turn upon strong noxious stimuli, which is rescued by AIB-specific expression of *inx-1*. It remains to be discovered what ion goes through the *inx-1* dependent gap junctions, or whether INX-1 forms gap junction or hemichannels to mediate the omega turn avoidance behavior (Fig. 2E).

### Current transfer in male-specific neural circuit

*Caenorhabditis elegans* males have sexually dimorphic connectivity that lead to male-specific behaviors (Cook et al., 2019; Jarrell et al., 2012). As such, males have about 400 and 350 additional chemical synapses and gap junctions, respectively, compared to hermaphrodites. In the copulation circuit in males, *unc-7*-dependent gap junctions dampen chemical synaptic input. UNC-7 forms gap junctions between the post cloacal sensilla neuron PCB and hook neuron HOA (Correa et al., 2015). PCB receives chemical synaptic input from PCA neurons. Optogenetic stimulation of PCA in *unc-7* mutant results in increased calcium levels in PCB, and ablation of HOA neurons also resulted in increased calcium levels in PCB (Correa et al., 2015). These results suggest that *unc-7* dependent gap junctions likely allow ion transfer from PCB to HOA to reduce and modulate PCB's calcium response upon chemical synaptic input from PCA (Fig. 2F).

### Rewiring of sensory neuron circuit by ectopic Connexin expression

ASEL and ASER are salt sensing neurons that are in close proximity but are not connected via chemical synapses or gap junctions. ASEL responds to increase in salt concentration, whereas ASER responds to decrease in salt concentration (Suzuki et al., 2008). As mentioned earlier, innexins and connexins share no scorable amino acid similarity, yet both function as gap junctions. To test directly whether ectopic expression of gap junction protein can change neural circuit connectivity, Rabinowitch et al. (2014) performed two sets of elegant experiments. Connexin 36 (Cx36) expression in ASEL and ASER neurons resulted in calcium responses in both neurons upon increasing or decreasing salt concentrations, demonstrating that ectopic expression of a single gap junction protein is sufficient to rewire neural connectivity. Similar expression of Cx36 in two other neurons, AWC and AIY neurons that are connected only via inhibitory chemical synapses, also resulted in gap junction formation that led to a switch from inhibitory to excitatory connectivity (Fig. 2G). Furthermore, ectopic expression of Cx36 in the CEP and RIH neurons was sufficient to induce artificial neuromodulation of nose touch response (Rabinowitch et al., 2013). In thermosensory neural circuit, AFD-AIY chemical synapse plasticity transforms thermosensory information and memory to temperature preference behavior (Hawk et al., 2018). Ectopic expression of Cx36 in both AFD and postsynaptic AIY neurons bypassed presynaptic plasticity in AFD and resulted in enhanced thermotaxis and thermosensory response in AIY regardless of temperature experience history. These findings demonstrate that changes in gap junction protein expression is sufficient for gap junction formation that can alter neural circuit connectivity and brain function.

## Innexins can also function independently of channel opening activity

In mammals, connexins are known to have functions independent of channels (Simonsen et al., 2014). Innexin hemichannel function in the *C. elegans* nervous system was first proposed by Yeh et al. (2009). Walker and Schafer (2020) recently provided definitive evidence that *unc-7* has gap junction-independent function as a hemichannel in mechanosensation. *unc-7* and *unc-9* are required for electrical coupling between ALM and AVM touch neurons. ALM and PVD neurons’ calcium responses to gentle and harsh touch, respectively, are impaired in *unc-7* null mutant. Both defects were rescued by expression of an *unc-7* mutant allele that lacks electrical coupling property due to the lack of four extracellular cysteines required for hemichannel interactions for gap junction formation (Bouhours et al., 2011). Despite no sequence similarity between innexins and connexins, the four extracellular cysteine positions are conserved. This is a direct evidence of a gap junction independent role of *unc-7* in mechanosensation. The authors further report that *unc-7* is responsible for the transient response to harsh touch in ALM neurons and is distinct from that of a different ion channel, *mec-4*, in the prolonged response to harsh touch. Ectopic expression of cysless-*unc-7* in olfactory neurons was sufficient to elicit nose-touch responses assayed by calcium responses and behavioral response to nose touch. The same innexin, *unc-7*, can have both channel and hemichannel functions in the same sensory neural circuit. Other innexins’ hemichannel functions in the nervous system, and how the gap junctional and junction-independent functions of innexins are determined remain open questions.

## Gap junctions play critical roles under stress conditions

All animals respond to stressful conditions and endure environmental changes. Recent findings in *C. elegans* also begin to uncover roles of innexins in the nervous system under altered environmental conditions. Under stress conditions such as sustained starvation, heat and high population density, developing *C. elegans* enter stress-resistant dauer stages (Fig. 2H). When food becomes available, dauers resume development into adults. Dauers have distinct morphology, metabolism and locomotion from non-dauers (Hu, 2007). Bhattacharya et al. (2019) characterized whether and how electrical synaptic connections differ in non-dauer and dauer worms in the nervous system, using fosmid-based reporter constructs for 15 innexin genes (17 including different isoforms). Many innexins such as *unc-7*, *unc-9*, *inx-1*, *inx-10* and *inx-14* are broadly expressed in the nervous system in both dauer and non-dauer animals. *inx-1b*, *che-7*, *inx-6*, *inx-7*, *inx-10a*, *inx-11*, *inx-14*, *inx-18a*, *unx-7* and *unc-9* have dauer-specific expression in at least one neuron type.

Among these, *inx-6* is expressed only in AIB neurons and only in dauer (Bhattacharya et al., 2019). EM reconstruction and colocalization studies show that AIB neurons form gap junctions with BAG neurons through INX-6 and INX-4 (CHE-7) (Fig. 2H). Loss of *inx-6* or *inx-4/che-7* causes a dauer-specific locomotion defect. This dauer-specific locomotory behavior was induced by ectopic expression of *inx-6* in AIB neurons in non-dauer animals. Several factors, such as transcription factors *unc-42* and *daf-16*, are required for *inx-6* upregulation, but how *inx-6* regulates the downstream pathway is still unknown. INX-6 is unique in that it can form hexadecameric gap junction channels (Oshima et al., 2013, 2016). *inx-6* shows higher stability in the lipid bilayer compared to the other innexins, along with higher permeability and larger pore size. Whether the unique properties of the structure account for the special role of *inx-6* in the dauer stage might be an intriguing question to study.

## Concluding remarks

Since the discovery of innexin genes in *C. elegans*, it has become evident that innexins are functionally equivalent to connexins. Like connexins, innexins regulate chemical synaptic localization, function as channels to regulate neural activity and as hemichannels in a sensory circuit, and are also involved in stress response. Innexins are expressed broadly and dynamically with cell specificity in the nervous system. Notably, *unc-7* and *unc-9*, which were proposed to function together based on phenotypic similarities, are now known to be required either together (as gap junction channels) or separately (as hemichannels) in synaptogenesis, locomotor circuit and mechanosensation. However, most other innexins do not seem to be as strongly paired as *unc-7* and *unc-9*, and can have redundant or partly overlapping function. Several open questions remain to be further addressed: (1) 17 innexins (including different isoforms) are expressed in neurons, six of which have been functionally characterized in the nervous system (Fig. 2), but the rest of the neuronally expressed innexins in the nervous system remain to be investigated. (2) In addition to forming gap junctions, some innexins function as hemichannels, but do other innexins function as hemichannels, and what determines whether an innexin forms junctions or hemichannels? Future studies await more detailed investigations of how innexins are differentially regulated when playing junctional and non-junctional roles. (3) How are gap junction and hemichannel localization, formation and the dynamic expressions of innexins regulated? Innexin localization can be interdependent, such as for *unc-7* and *unc-9*, and *inx-18* regulates *inx-19* localization. Other regulators of innexin expression and gap junction formation remain to be further investigated. (4) In other organisms including *Drosophila*, gap junctions and hemichannels can function as heteromeric channels that are composed of a mixture of innexins (Phelan and Starich, 2001). The precise channel compositions for most *C. elegans* innexins remain to be determined. (5) Maintenance of chemical synapses is important for neuronal function and health, and defects in cellular maintenance mechanisms, such as chemical synaptic protein turnover, can lead to neurodegeneration. How gap junctions and gap junction proteins are turned over in a healthy brain, and how gap junction plasticity is regulated and maintained remain open questions. Whether these mechanisms are coupled with the maintenance mechanisms for chemical synapses would further our fundamental understanding of how chemical and electrical synapses coordinate brain development and function.
